# Effect of non-linearity of a predictor on the shape and magnitude of its receiver-operating-characteristic curve in predicting a binary outcome

**DOI:** 10.1038/s41598-017-10408-9

**Published:** 2017-08-31

**Authors:** Kwok M. Ho

**Affiliations:** 1 Department of Intensive Care Medicine, Royal Perth Hospital, Perth, Australia; 2 School of Population Health, University of Western Australia, Perth, Australia; 3 School of Veterinary & Life Science, Murdoch University, Perth, Australia

## Abstract

Area under a receiver-operating-characteristic (AUROC) curve is widely used in medicine to summarize the ability of a continuous predictive marker to predict a binary outcome. This study illustrated how a U-shaped or inverted U-shaped continuous predictor would affect the shape and magnitude of its AUROC curve in predicting a binary outcome by comparing the ROC curves of the worst first 24-hour arterial pH values of 9549 consecutive critically ill patients in predicting hospital mortality before and after centering the predictor by its mean or median. A simulation dataset with an inverted U-shaped predictor was used to assess how this would affect the shape and magnitude of the AUROC curve. An asymmetrical U-shaped relationship between pH and hospital mortality, resulting in an inverse-sigmoidal ROC curve, was observed. The AUROC substantially increased after centering the predictor by its mean (0.611 vs 0.722, difference = 0.111, 95% confidence interval [CI] 0.087–0.135), and was further improved after centering by its median (0.611 vs 0.745, difference = 0.133, 95%CI 0.110–0.157). A sigmoidal-shaped ROC curve was observed for an inverted U-shaped predictor. In summary, a non-linear predictor can result in a biphasic-shaped ROC curve; and centering the predictor can reduce its bias towards null predictive ability.

## Introduction

Area under a receiver-operating-characteristic (AUROC) curve is widely used in medicine to summarize the ability of a continuous diagnostic or predictive marker to diagnose or predict a binary or dichotomized outcome, such as with and without a disease, respectively^1^. In addition, AUROC derived from a regression model has also been used, with caveats, to reflect the performance of a regression model^2^. Using ‘ROC curve’, ‘receiver operating characteristic curve’ or ‘AUROC’ as the search terms yielded a total of 64,212 returns from the PUBMED database (on March 28, 2017), suggesting that this summary statistic was often reported in the medical literature.

The ROC curve is constructed by plotting true positive rates (or sensitivity) against the false positive rates (or 1-specificity) using different cut-off levels for a continuous predictor to predict a binary outcome. Each point on the curve thus represents a balance, or trade-off, between sensitivity and specificity for a certain diagnostic or prognostic threshold for the continuous predictor; and the summation of the area under the curve formed by each of the diagnostic cut-points is the AUROC. A test with perfect discrimination will have an AUROC curve equals to one, with the curve passes through the upper left corner with 100% sensitivity and 100% specificity. The AUROC curve is very useful because it quantifies the discriminative ability of a continuous predictor and is not affected by the prevalence of the disease^3, 4^, making the result of this diagnostic test more likely to be comparable across different patient populations with different prevalence of the outcome. Depending on the relative importance of sensitivity and specificity, we can also use the ROC curve to define the optimal cut-off level for a diagnostic or prognostic test^3, 5^. Finally, the likelihood ratio for a single diagnostic test value can be easily measured from the slope of the tangent of the curve represented by that point^6^.

Based on nonparametric Mann-Whitney U statistics in calculating the AUROC curve, we can interpret the AUROC as the probability that a randomly selected patient with an outcome is associated with a higher value in the predictor under investigation than that for a randomly chosen individual without the same outcome^7^. This can be visualized by assessing the concordance between the observed outcome (as 0 or 1 on the x-axis) and predictor’s values (or the predictor’s derived risks of the outcome on the y-axis). The probability of the slope of these concordance lines being positive is the AUROC.

AUROC can also be calculated by the following formula: (http://www.cs.ru.nl/~tomh/onderwijs/dm/dm_files/ROC101.pdf):

$${\rm{AUROC}}=(1/{\rm{mn}})\,{\rm{x}}\,\sum _{i=1}^{{\rm{m}}}\sum _{j=1}^{{\rm{n}}}1(\mathrm{if}\,\mathrm{Pi} > \mathrm{Pj})$$

where the ‘m’ is the number of predictor values associated with the presence of an observed outcome and ‘n’ is the number of predictor values without the observed outcome; and P_*i*_ and P_*j*_ denote the predictor’s values assigned to the data points *i* and *j*, respectively.

As such, for a continuous predictor with a bimodal or U-shaped association with an outcome, it is likely that the AUROC curve will be biased towards a null association. The co-existence of negative and positive concordance will cancel out, at least in part, the predictive effect of the positive concordance. In many biological systems, a risk factor or physiological derangement may have a U-shaped (with a bimodal concordance) or non-linear effect on different patient outcomes, including arterial carbon dioxide tension, temperature, white blood cell count, plasma potassium or even body mass index^8–10^. For instance, it is well established that patients admitted to the critical care unit with either severe hypothermia or hyperthermia, and leucopenia or leucocytosis are associated with a higher risk of death than those with a normal temperature and white blood cell count, respectively.

Although the importance of transforming a non-linear predictor in a linear or logistic multivariate regression has been well known, how a U-shaped continuous predictor would affect the shape and magnitude of a ROC curve in predicting a binary or dichotomized outcome in a univariate analysis in clinical settings have not been well described^11^. Using actual patient data, this study aimed to illustrate how a non-linear U-shaped and inverted U-shaped continuous predictor would affect the shape and also magnitude of the AUROC curve in predicting a binary or dichotomized outcome.

## Methods

All patients who were admitted to the Royal Perth Hospital Intensive Care Unit (ICU) between 1st January 2008 and 31 December 2013 were included in this study, excluding those who were readmitted during the same hospitalization^12^. Royal Perth Hospital is a 450-bed university teaching hospital and the 20-bed tertiary ICU admits critically ill adult patients of all specialties except cardiothoracic surgery and liver transplantation. During the study period, all the components of the Acute Physiology and Chronic Health Evaluation (APACHE) II score including the worst first 24-hour physiology and biochemical data were recorded for all patients. After the patient was discharged from the ICU, the data were checked for transcription errors and completeness by a designated trained clerical staff member using data from the computerized laboratory database and going through the ICU vital signs flow chart again before the data were transferred to the computer. A single data-custodian has been responsible for ensuring data quality. The performance of the APACHE II score relative to other prognostic models using this dataset was described in our previous publication^13^. The administrative data used for this retrospective cohort study were collected for quality assurance purposes, and further analysis of the non-identifiable data was registered as a clinical audit with the Clinical Safety and Quality Unit (150521–02) and was exempt from review by the Royal Perth Hospital Ethics committee.

Our previous study showed that intensive care admission arterial pH was associated with a U-shape relationship with subsequent hospital mortality^14^. Because the worst first 24-hour physiology data is known to predict mortality better than admission physiology^12^, we used the worst first 24-hour arterial pH to assess its ability to predict hospital mortality with and without considering its U-shaped relationship with hospital mortality.

First, we assessed the AUROC of the worst first 24-hour arterial pH to predict mortality without centering or transforming the pH data. The shape of this ROC was inspected to assess whether this was different from the usual concave-downward shaped ROC curve^1, 3^. Second, we repeated the AUROC analyses after centering the pH by its mean (=7.34) or median (=7.36) values using the following equation (pH–mean pH)^2^ or (pH – median pH)^2^, respectively. This centering technique will mathematically convert a U-shaped curve into a relatively straight line. The ROC curves for these two centered pH variables were constructed to assess whether this was different from the ROC curve of pH without centering. The difference in AUROC derived from the same cases was calculated using the method suggested by Hanley and McNeil^15^. Finally, we assessed the possible effect of an inverted U-shaped predictor on the shape of the ROC curve. Although an inverted U-shaped relationship between a clinical predictor and adverse outcome has been observed in biological systems - for example, between severity of injury and fatal pulmonary embolism after major trauma^16^ – it is still relatively uncommon. For ease of illustration on how an inverted U-shaped predictor would affect the shape and magnitude of the ROC curve in this study, an arbitrary and hypothetical, non-randomly created, dataset of 500 subjects with an inverted U-shaped association between a continuous predictor and an outcome was constructed, assuming that both extremely high and low values of this predictor was associated with a lower risk of the outcome. The situation would be similar to using the same clinical dataset to assess the predictive ability of pH in relation to survival (instead of mortality) as an outcome.

As a sensitivity analysis, we also transformed the arterial pH by either a (natural) logarithm function or simply squaring of the pH values without centering, and assessed whether these transformations would improve the AUROC and change the shape of the ROC curve. No other covariates were added to pH in predicting mortality in all the analyses conducted in this study. That is, all AUROC results were based on univariate predictive values of pH, and pH alone. All statistical analyses were performed by SPSS for Windows (version 23.0, IBM, USA) and MedCalc for Windows (version 12.5, Ostend, Belgium), and a p-value < 0.05 was taken as significant in this study. The non-identifiable dataset used in this study will be available to the readers by contacting the corresponding author.

### Ethics approval and consent to participate

The administrative data used for this study were collected for quality assurance purposes, and further analysis of the non-identifiable data was registered as a clinical audit with the Clinical Safety and Quality Unit (150521–02) and was exempt from review by the Royal Perth Hospital Ethics committee.

## Results

**Figure 1:**
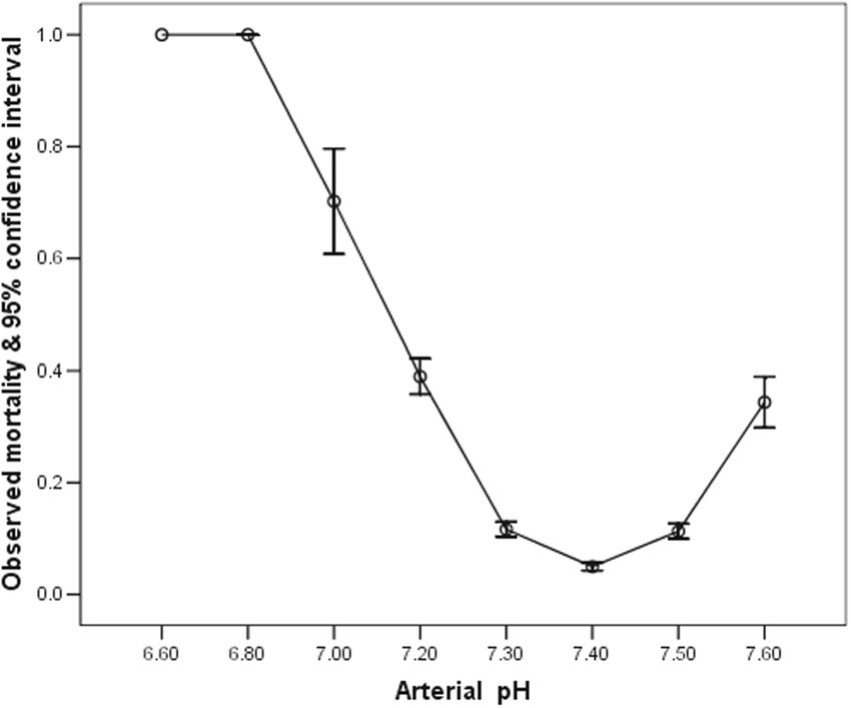
Relationship between the worst first 24-hour arterial pH and hospital mortality.

**Figure 2:**
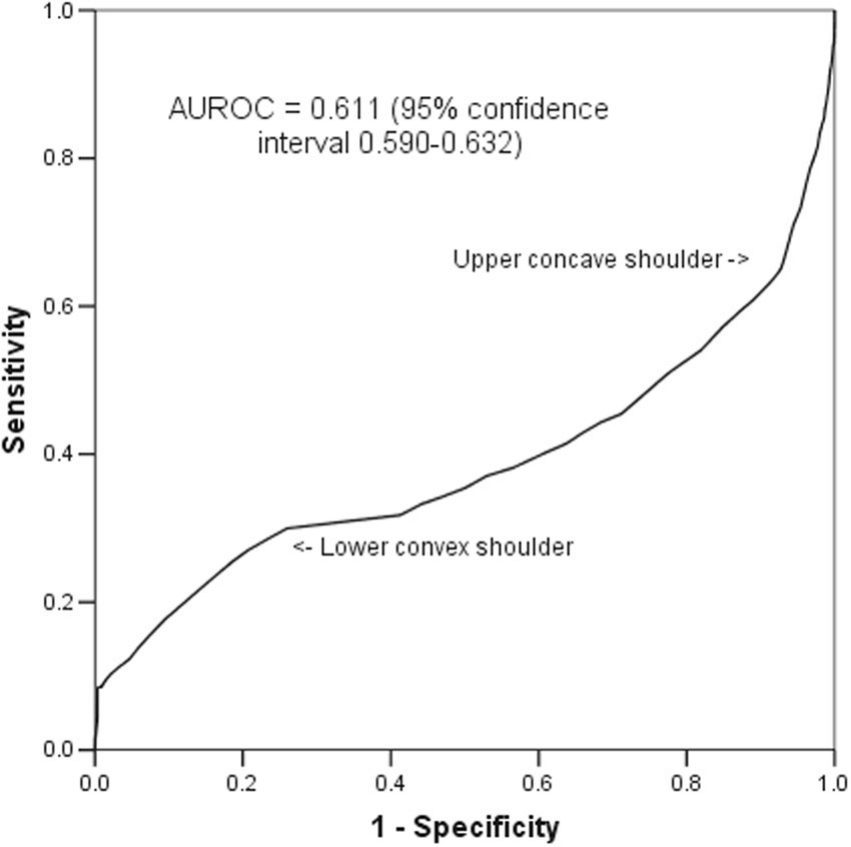
Receiver operating characteristic curve of arterial pH without centering in discriminating between hospital survivors and non-survivors.

The mean and median of the worst first 24-hour arterial pH of 9549 consecutive patients were 7.34 and 7.36, respectively (range: 1.4, interquartile range: 7.28–7.41) and it had an asymmetrical U-shaped relationship with hospital mortality (Fig. 1). The AUROC of the arterial pH, without centering, to discriminate between survivors and non-survivors was 0.389 (95% confidence interval [CI] 0.368–0.410), which was equivalent to 0.611 (95%CI 0.590–0.632) if we considered the inverse relationship between an increased risk of death and decreasing pH. The ROC curve of this asymmetrical predictor had a ‘lower shoulder’ and an ‘upper shoulder’ similar to a logit or inverse-sigmoidal curve (Fig. 2); and this was quite different from the usual concave-downward shaped ROC curve^1, 3^.

**Figure 3:**
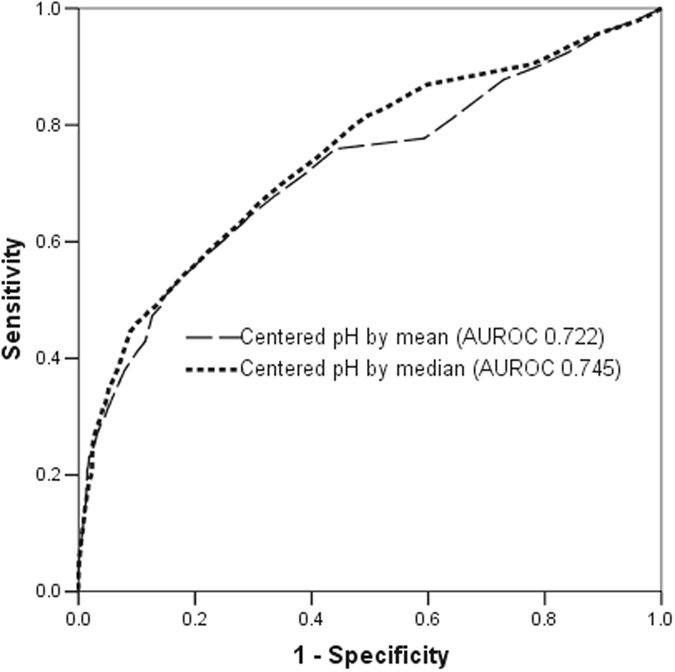
Receiver operating characteristic curve of arterial pH after centering by its mean or median in discriminating between hospital survivors and non-survivors.

The AUROC significantly increased after centering the pH by its mean (0.611 vs 0.722, difference = 0.111, 95% confidence interval [CI] 0.087–0.135), and this was further improved after centering the predictor by its median (0.611 vs 0.745, difference = 0.133, 95%CI 0.110–0.157)(Table 1). The ROC curves of these two centered pH variables were much more similar to the usual concave downward-shaped ROC curve (Fig. 3).

**Table 1:** Area under the receiver operating characteristic (AUROC) curve of arterial pH with and without centering in predicting hospital mortality of the critically ill.

	AUROC (95% confidence interval [CI])	Difference in AUROC compared to pH without centering (95%CI)	P value
(1) pH without centering	0.611 (0.590–0.632)^*^		
(2) pH after centering by its mean	0.722 (0.705–0.739)	0.111 (0.087–0.135)	0.001
(3) pH after centering by its median	0.745 (0.729–0.760)^#^	0.133 (0.110–0.157)	0.00

NB: ^*^The AUROC for pH without centering was generated after considering the inverse relationship between increased risk of death and decreasing pH; the AUROC without considering this inverse relationship was 0.389 (95%CI 0.368–0.410), and AUROC did not change after logarithm transformation or squaring the pH. ^#^The difference in AUROC between pH after centered by its mean and pH after centered by its median was 0.023 (95%CI 0.015–0.030, p = 0.001).

**Figure 4:**
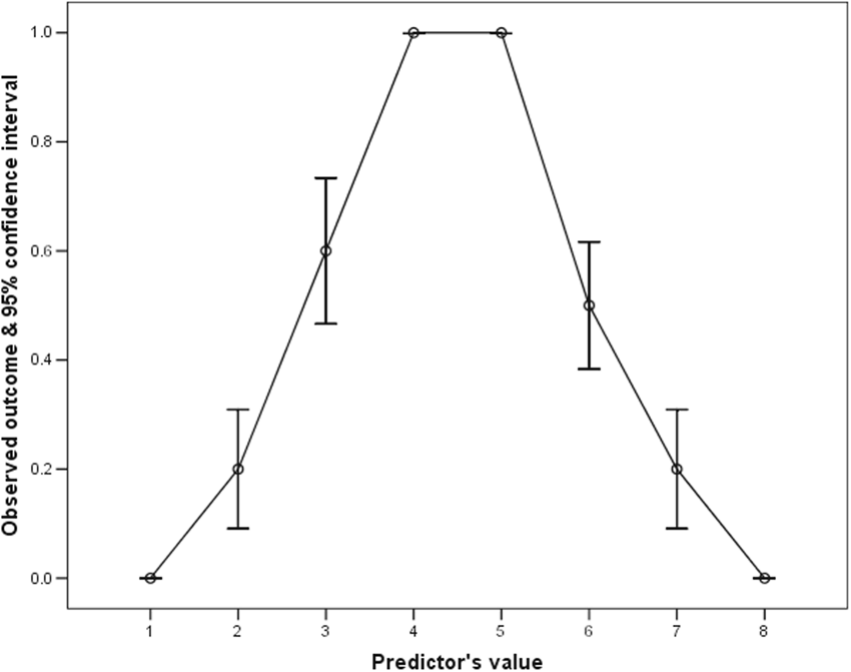
An inverted U-shaped relationship between an arbitrary created predictor and its outcome.

**Figure 5:**
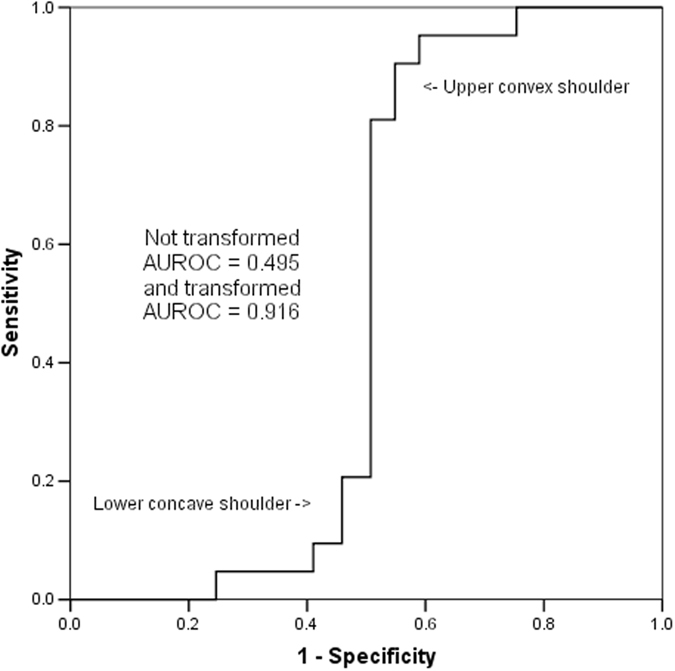
A sigmoidal-shaped receiver operating characteristic curve of an arbitrary created U-shaped continuous predictor.

With a simulated symmetrically inverted U-shaped predictor (Fig. 4), the shape of the ROC curve became a sigmoidal-shaped (Fig. 5) and centering of this predictor by its mean also substantially improved the AUROC (0.495 vs 0.916).

In the sensitivity analysis, the inverse-sigmoidal (or logit) shape of the ROC curve and AUROC (0.611, 95%CI 0.590–0.632) both did not change after logarithmic transformation and simply squaring of the pH values without centering.

## Discussions

This study showed that a substantial non-linear relationship between a continuous predictor and a binary outcome can distort the shape of a ROC curve. Depending on whether it is a U-shaped or inverted U-shaped predictor, the ROC may take the form of inverse-sigmoidal or sigmoidal shape, respectively. In addition to affecting the shape of the ROC curve, a U-shaped non-linearity also has a tendency to induce a bias toward null association (or AUROC = 0.5). Our results suggest that centering an asymmetrical U-shaped predictor by its median is better than centering by its mean in improving the effect of non-linearity, resulting in a better improvement in AUROC. These results have significance and require careful consideration.

**Figure 6:**
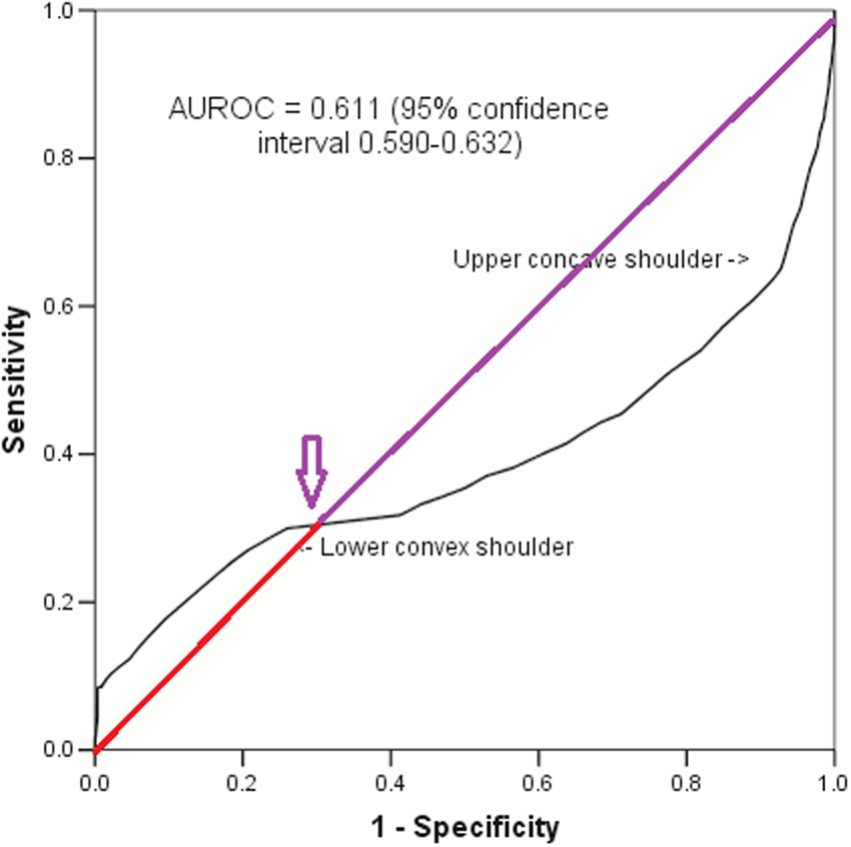
Intersection of the diagonal reference line and the inverse-sigmoidal curve of pH in predicting mortality of the critically ill.

First, our results suggest that visual inspection of the ROC may provide some information of the possibility of non-linearity of a continuous predictor in predicting a binary outcome. As such, it would be prudent to inspect the shape of the ROC curve when non-linearity of a predictor is suspected or biologically plausible^7–9, 16^, in addition to reporting the value of AUROC and the associated 95% confidence interval. An inverse-sigmoidal or sigmoidal-shaped ROC curve also implies that the curve will intersect with the straight diagonal reference line at a certain point on the ROC plot, as shown by the arrow in Fig. 6. The slope of a straight line from point (0,0) to this specific intersection point defines the positive likelihood ratio (LR (+)) of the test at this point which is equal to one^6^. Conversely, the slope of a straight line from point (1,1) to this intersection point defines the negative likelihood ratio (LR (−)) of the test at this point which is also equal to one^6^. The portion of the ROC curve above the straight diagonal line suggests the test is useful with a LR (+) >1 and a LR (−) <1; whilst the portion of the ROC curve below the straight diagonal line is also useful, but in an inverse fashion, with a LR (+) <1 and a LR (−) >1. The latter implies that the test results have an inverse relationship to the chance of observing the outcome. The intersection point between the ROC curve and the straight diagonal reference line on the ROC plot corresponds to the nadir of the U-shaped curve or peak of the inverted U-shaped curve in relation to the risk of the binary outcome; and without centering the diagnostic test results, using this cut-point would offer no discriminative utility (or no predictive value beyond chance alone).

Second, when non-linearity between the outcome and continuous predictor is suspected or confirmed by the examination of the raw data, it will be advisable to consider centering the predictor before assessing the predictor’s AUROC. Without centering a substantially non-linear U-shaped predictor before ROC analysis may result in a bias toward null association (or prediction). Our results suggest that centering the predictor by its median is more effective than centering by its mean for an asymmetrical U-shaped predictor, while logarithmic transformation and simply squaring the predictor are not helpful. In some ways, a reduced AUROC of a non-linear predictor without centering is similar to a reduced statistical significance of a non-linear continuous predictor in a multivariate regression, when categorization at appropriate cut-points or a restricted cubic spline function is not used for the non-linear predictor^4^.

Third, AUROC can be constructed by either a parametric (assuming the test results follow a binormal distribution, or both discriminating groups have normal distributions using maximum likelihood estimation) or non-parametric method^17, 18^. The nonparametric estimate of AUROC curve is the summation of the areas of the trapezoids formed by connecting the points on the ROC curve. The nonparametric estimate of AUROC may underestimate AUROC for discrete rating data compared to a parametric method, but theoretically, both methods would not be able to overcome the potential bias towards null prediction (AUROC = 0.5) for a non-linear U-shaped predictor.

This study has some limitations. According to the statistical basis of ROC curve^7^, the more symmetrical the U-shaped relationship between the predictor and the outcome or the stronger the predictor it is (as evidenced by a steeper U-shaped curve), the stronger the bias towards a null predictive effect is expected by not centering the non-linear U-shaped predictor. Although we had used real clinical data to illustrate how a non-linear predictor can affect the shape and magnitude of a ROC curve, whether this effect is equally important to other non-linear clinical predictors, especially when the U-shaped is not as strong as arterial pH, remains uncertain. Even without transformation, most other forms of non-linear predictors, including those with a sigmoidal relationship to an outcome, should at least theoretically have less effect on the shape and magnitude of their ROC curves than a symmetrical U-shaped predictor; as most forms of non-linearity will not disrupt the (unimodal) concordance relationship between the probability of the observed outcome and the magnitude of the non-linear predictor’s values. Finally, we only used either the mean or median to center the non-linear predictor in this study. Whether simply assigning the nadir of U-shaped curve as normal and any values of the predictor below this nadir as having the same significance as the corresponding higher values above the nadir of the curve is better than centering by the predictor’s mean or median would depend on the precise form (or shape) of the non-linearity of the predictor under investigation.

In summary, a U-shaped or inverted U-shaped continuous predictor for a dichotomized outcome may create a logit (inverse-sigmoidal) or sigmoidal shaped ROC curve, respectively; and without centering the predictor, this may create a bias towards null prediction. Centering an asymmetrical U-shaped predictor by its median value results in a better improvement in its AUROC than by logarithm transformation or simply squaring the predictor’s values.
